# Investigation of a Human Case of *Francisella tularensis* Infection, United Kingdom, 2023

**DOI:** 10.3201/eid3010.240479

**Published:** 2024-10

**Authors:** Ameeka Thompson, Tim Brooks, Catherine Houlihan, Tommy Rampling, Helen Umpleby, Kayleigh Hansford, Jolyon Medlock, Alexander Vaux, Julie Logan, Andrew Frost, Sue Neale, Stephen Wyllie, Kirsty Dodgson, Dominic Haigh, Isra Halim, Raqib Huq, Michael Riste, N. Claire Gordon

**Affiliations:** UK Health Security Agency Rare and Imported Pathogens Laboratory, Salisbury, UK (A. Thompson, T. Brooks, C. Houlihan, T. Rampling, H. Umpleby, N.C. Gordon);; UK Health Security Agency Medical Entomology and Zoonoses Ecology, Salisbury (K. Hansford, J. Medlock, A. Vaux);; UK Health Security Agency Bacterial Identification Section, London, UK (J. Logan);; Animal and Plant Health Agency Veterinary Advice Services, London (A. Frost);; Animal and Plant Health Agency Surveillance and Laboratory Service Department, Cumbria, UK (S. Neale);; Animal and Plant Health Agency Veterinary Advice Services, Surrey, UK (S. Wyllie);; Manchester University NHS Foundation Trust, Manchester, UK (K. Dodgson, D. Haigh, I. Halim, R. Huq, M. Riste)

**Keywords:** *Francisella tularensis*, zoonoses, tularemia, bacteria, bacterial zoonoses, United Kingdom

## Abstract

Tularemia, caused by *Francisella tularensis*, is not known to occur in the United Kingdom. We report a case of tularemia diagnosed in July 2023 in a UK patient with no travel in the 6 weeks before symptom onset. We describe the subsequent multiagency investigation into possible routes of acquisition.

Tularemia is a zoonotic disease caused by *Franciscella tularensis*, currently considered absent from the United Kingdom. We report a human case of *F. tularensis* subspecies *holarctica* infection diagnosed in the United Kingdom and the resulting cross-agency investigations. We obtained written consent from the patient for publication of our findings. 

## The Study

In May 2023, a 47-year-old man sought treatment at a primary care facility 48 hours after he had fever and cervical lymphadenopathy develop. The working diagnosis was a dental infection, but there was no response to the oral antimicrobial drugs amoxicillin/clavulanic acid and metronidazole or to subsequent dental extraction. The patient was referred to the otorhinolaryngology department for further investigation. Over the next few weeks, the lymph nodes became suppurative, but there was no growth on routine bacterial culture. We aspirated his lymph nodes in June 2023 but found no growth on bacterial or mycobacterial cultures and sent the sample for 16S rRNA gene PCR (16S PCR) and sequencing. *F. tularensis* was identified on 16S PCR and confirmed by subsequent *Francisella*-specific PCR at the UK Health Security Agency (UKHSA) Rare and Imported Pathogens Laboratory. 

We initially assumed that the infection had been acquired during a trip to Sweden. However, after the *F. tularensis* diagnosis was confirmed, we obtained a detailed travel and exposure history from the patient. The patient’s symptoms had manifested 6 weeks before the trip to Sweden and remained unchanged after the trip. His only other relevant overseas travel was a 4-day trip to southern Portugal in March 2023, >6 weeks before symptom onset. Before that time, he had not left the United Kingdom since 2019.

The patient had 3 pet degus, chinchilla-like rodents originating in Chile, bought in the United Kingdom 6 years earlier. One degu had died suddenly in early July 2023, two months after the patient’s symptom onset. The corpse of the animal was disposed of without examination. A second degu became unwell with jaw swelling a few days later. Cytological examination of the swelling was in keeping with inflammation and infection, but no samples were sent for bacteriology. The patient reported no other animal contact apart from occasional contact with dogs of a family member. Those dogs were fed commercial nonraw pet food and had had no recent illnesses.

The patient lived near a nature reserve in the northwest of England where he took regular walks; he had not seen any lagomorphs or rodents or noticed any insect bites. He had no history of drinking contaminated water or consuming raw meat, lagomorphs, or rodents and had not handled any animal carcasses. The patient was examined again by the local infection team. Because of ongoing discharge from the neck lesions, antimicrobial drugs were changed from doxycycline to ciprofloxacin. 

We collected additional swab and serum samples for *F. tularensis* antibody testing. *F. tularensis* subsp. *holarctica* subtype was confirmed by specific PCR by using an in-house multitarget real-time TaqMan PCR differentiation assay developed as described elsewhere (*1*). We submitted the PCR product for next-generation sequencing, which confirmed subspecies identification. Unfortunately, we could not culture the organism from either the original or later swab samples. A commercial assay (Seramun Diagnostica, https://www.seramun.com) was positive for combined *F. tularensis* IgG/IgM. We took samples from the surviving degus and their bedding and food. All tested negative for *F. tularensis* by specific PCR, and the bacterium was not isolated on culture. 

The United Kingdom has many potential mammal reservoirs of *F. tularensis*, including the brown hare (*Lepus europaeus*), black rat (*Rattus rattus*), and several mice and vole species, but the organism has never been isolated from animals in the United Kingdom. The Animal and Plant Health Agency through its Diseases of Wildlife Scheme conducts passive surveillance for wildlife diseases; no local wildlife dieoffs had been reported. Rewilding programs reintroducing beavers (*Castor fiber*) into the United Kingdom present another possible source; however, no beavers had been released near the patient’s locale, and imported beavers must test negative for *F. tularensis* as a condition of release. 

Tickborne transmission of *F. tularensis* subsp. *holarctica* has been described for *Ixodes ricinus* and *Dermacentor reticulatus* ticks, both present in the United Kingdom. Although the likely date of the patient’s tularemia acquisition was compatible with seasonal activity of *I. ricinus* ticks, there were no records of that tick in the local area, based on 15 years of passive surveillance data (*2*). Similarly, *D. reticulatus* ticks are not known to be present in that area. Mosquitoes represent another potential vector. Data from national mosquito surveillance indicate that potential mosquito vectors, such as *Aedes cinereus*, are present in the United Kingdom but would have been active 2 weeks after symptom onset. No nuisance biting was reported in the area at that time.

Site visits were conducted by the UKHSA Medical Entomology and Zoonoses Ecology team in August 2023 and May 2024. We found no evidence of active tick populations after sampling. Mosquito traps did not yield any human-biting species. 

Although the patient’s travel dates made acquisition in Portugal highly unlikely, we notified authorities in that country using its National International Health Regulations Focal Point in accordance with the World Health Organization International Health Regulations (2005) (*3*). The Portugal authorities have reported no recent imported or autochthonous cases of tularemia. European Centre for Disease Prevention and Control tick surveillance data show that both *I. ricinus* and *D. reticulatus* ticks are present in southern Portugal (*4*,*5*). A surveillance study project conducted in northern Portugal detected *F. tularensis* DNA in 1 human and 1 tick (*6*). Both imported (*7*) and autochthonous (*8*) cases of tularemia have been described in Portugal, although the autochthonous case was diagnosed by agglutination testing only. If the patient did acquire the infection in Portugal, the incubation period would have been substantially longer than usual because he returned from Portugal 47 days before symptom onset (*9*). The longest incubation period reported is 43 days in 1 case report (*10*). 

Several hypotheses have been proposed and investigated to explain the transmission route for the case-patient. Whereas acquisition in Portugal was a possibility, the unusually long incubation period and reported local absence of *F. tularensis* in both humans and wildlife makes that scenario unlikely. Similarly, the patient was unlikely to have acquired the infection from his companion animals given the timelines and negative testing of the animals. We cannot rule out acquisition within the United Kingdom, potentially from an as-yet unidentified environmental reservoir, with the possibility that it was transmitted by a biting arthropod. Following antimicrobial treatment, the patient’s systemic symptoms resolved. The lymph nodes ceased suppurating but remained enlarged, although without pain. 

## Conclusions

UKHSA issued a briefing note (*11*) to inform clinicians of the possible presence of *F. tularensis* in the United Kingdom. Passive surveillance for reports of wildlife dieoffs in the area will continue, and the nature reserve will be incorporated into a nationwide mosquito surveillance project. *F. tularensis* testing will be conducted on ticks collected as part of ongoing surveillance and research. 

This multidisciplinary investigation demonstrates the difficulties of establishing the origin of a cryptic infection. Clinicians should be aware of tularemia and consider it in differential diagnoses of patients with compatible clinical syndromes, even in the absence of a recent relevant history of travel. 
